# Meniscal Bucket-Handle Tears and Return to Sports in Young Adults: A Systematic Review and Meta-Analysis

**DOI:** 10.7759/cureus.81329

**Published:** 2025-03-28

**Authors:** Vasiliki Tsakiri, Theofylaktos Kyriakidis, Polychronis Papadopoulos, Vasileios Davitis, Byron Chalidis, Periklis Papadopoulos, Ioannis Gigis

**Affiliations:** 1 2nd Orthopaedic Department, General Hospital "G. Gennimatas" Aristotle University of Thessaloniki, Thessaloniki, GRC; 2 School of Medicine, 1st Orthopaedic Department, Aristotle University of Thessaloniki, Thessaloniki, GRC

**Keywords:** bucket-handle tears, meniscal repair, meniscal sutures, return to sports, young patients

## Abstract

Bucket-handle tears represent a subtype of meniscal injuries that involve a full-thickness longitudinal tear. These tears are challenging and demanding. The present systematic review and meta-analysis aimed to evaluate return-to-sport outcomes in young adults with isolated bucket-handle meniscal tears treated surgically through meniscal repair. This review followed the Preferred Reporting Items for Systematic Reviews and Meta-Analyses (PRISMA) guidelines, with two independent reviewers selecting studies from PubMed, Web of Science, and Scopus databases. Studies were included if they involved patients over 16 years old, with isolated bucket-handle tears, a minimum of one-year follow-up, and reported postoperative Tegner scores. Five studies comprising 168 individual knees met these criteria and were analyzed. Quality assessment employed the Newcastle-Ottawa Scale. Data analysis was conducted in R (R Foundation for Statistical Computing, Vienna, Austria) for pooled outcome calculations. The primary outcome was the postoperative Tegner score, representing a return to sports; secondary outcomes included changes in Tegner and Lysholm scores and failure rates. Two cohort studies and three case series were included, with quality ratings ranging from fair to poor. The mean patient age was 27.8 years, and the mean follow-up was 82.4 months. The pooled postoperative Tegner score was 5.94 (95% CI: 5.41-6.46), indicating a high return to recreational sports. The mean change in Tegner score was 2.48 (p = 0.0604), which was not statistically significant, while Lysholm score improvement was significant at 31.16 points (p = 0.0093). The pooled failure rate across studies was 14% (95% CI: 0-42%). Patients with isolated bucket-handle meniscal tears undergoing surgical repair demonstrate a high rate of return to recreational sports, with significant improvements in Lysholm scores. However, failure rates and study quality variability suggest further high-quality research to make safer conclusions.

## Introduction and background

The menisci play a crucial biomechanical role in the normal functioning of the knee, including load distribution, shock absorption, and joint stabilization [1,2]. They help reduce overall contact stress by increasing the contact area within the knee, thereby protecting the articular cartilage from mechanical damage. They also contribute to nutrition, joint lubrication, and proprioception [3,4]. Bucket-handle tears represent a subtype of meniscal injuries that involve a full-thickness longitudinal tear, with a displacement of the central half of the meniscus into the intercondylar notch [5,6].

Isolated bucket-handle tears are more common in the medial meniscus due to their strong attachments to the joint capsule. Usually, they tend to occur in the peripheral two-thirds of the meniscus. This location coincides with the "red-red" and "red-white" vascular zones. They constitute about 10% of all meniscal tears and can be challenging to treat due to high reported failure rates [7-9].

The management of meniscal tears has evolved significantly during the last years, following a better understanding of the physical history of knee degeneration and quicker development of osteoarthritis after losing a part of the meniscus. Therefore, there has been a shift toward preserving the meniscus as the first line of treatment [10,11].

Several repair techniques have been described in the literature, with all-inside repair gaining popularity during recent years over the "gold-standard" inside-out and the outside-in technique, as well as various hybrid techniques combining the above. All-inside repair techniques demonstrate equal functional outcomes and healing rates, are quicker, minimally invasive, less technically challenging, have faster recovery, and fewer complications. Existing studies frequently report outcomes of bucket-handle meniscal tears (BHMTs) in cohorts that include patients with concomitant anterior cruciate ligament (ACL) injuries, limiting their applicability to isolated cases. ACL could be a confounder, as ligament instability and its surgical reconstruction can significantly impact rehabilitation protocols and functional outcomes [12-14].

To draw definitive conclusions about the prognosis of isolated BHMTs is therefore tricky, particularly in the population of recreational athletes. This study explores the high number of recreational athletes who participate in sports for enjoyment and social relations. This population is continually increasing due to changes in lifestyle habits. For these individuals, understanding the rates of return to pre-injury activity levels and the expected timeline for recovery is crucial for setting realistic expectations and guiding clinical decision-making. This systematic review and meta-analysis aims to evaluate return-to-sport outcomes in young adults with isolated BHMTs.

## Review

Materials and methods

Study Type and Search Strategy

This systematic review and meta-analysis was conducted in accordance with the Preferred Reporting Items for Systematic Reviews and Meta-Analyses (PRISMA) guidelines. The PubMed, Web of Science, and Scopus databases were used as data sources for available studies from 2000 until the 30th of June 2024. The keywords “meniscus”, “bucket-handle” or “BH” or “bucket handle”, “outcomes” or “outcome”, as well as “return to sport” were used, combined with the Boolean operators “AND” and “OR”.

Eligibility Criteria

The inclusion criteria for all the eligible studies were the following: (1) articles published in the last 20 years; (2) minimum follow-up of one year; (3) primary bucket-handle injury treated with surgical repair; (4) studies including report of the postoperative Tegner score; (5) studies including the non-professional athlete population of over 16 years; (6) articles published in English, French, or German.

Exclusion criteria include the following: (1) studies including ACL reconstructed knees in the same cohort when reporting the postoperative Tegner score; (2) non-human studies; (3) studies including patients less than 16 years old; (4) studies with incomplete data; (5) systematic reviews and meta-analyses.

Data Extraction

Patient demographics, follow-up periods, tear zone, type of treatment, surgical technique and rehabilitation, failure rate, and outcome assessment scores were compiled. In all but one of the eligible studies, the outcome data were extracted as patient subgroups of a larger cohort, which contained patients with an isolated bucket-handle injury in stable knees. The rest of the patients included in those cohorts had sustained bucket-handle tears along with ACL rupture and were therefore not included in this review and meta-analysis. In one study, data were directly extracted from the patients’ dataset [15].

Quality and Risk of Bias Assessment

To determine the quality of the selected studies, a modified Newcastle-Ottawa Scale (NOS) for cohort studies was used, which is considered a valid tool for non-randomized studies [16]. The NOS uses a star system and consists of three separate sectors in quality assessment: selection, comparison, and outcome. The stars are attributed according to the answer to the questions of each sector. According to the stars given, the study is assessed as of “Good”, “Fair”, or “Poor” quality.

Statistical Analysis

Data from the selected studies were analyzed using the R software (R Foundation for Statistical Computing, Vienna, Austria) and RStudio (Posit, Boston, MA). The “meta” package and its various functions were deployed for this purpose. The primary outcome of this study was the postoperative Tegner score, as a quantitative representation of return to sports. Secondary outcomes included the change in Tegner score, the change in Lysholm score, and the failure rate. Heterogeneity between studies was assessed by performing the Higgins and Thompson I^2^ heterogeneity test. DerSimonian and Laird’s random-effects model was chosen for the meta-analysis, as it was assumed that the estimated effect size could vary between studies not only due to sampling error, but also due to other differences among the studies [17], like the different surgical techniques used, different rehabilitation protocols, and other possible differences in treatment and/or patient characteristics. Thus, it was assumed that this model would provide a more realistic estimate of the effect size.

Results

Search Results

**Figure 1:**
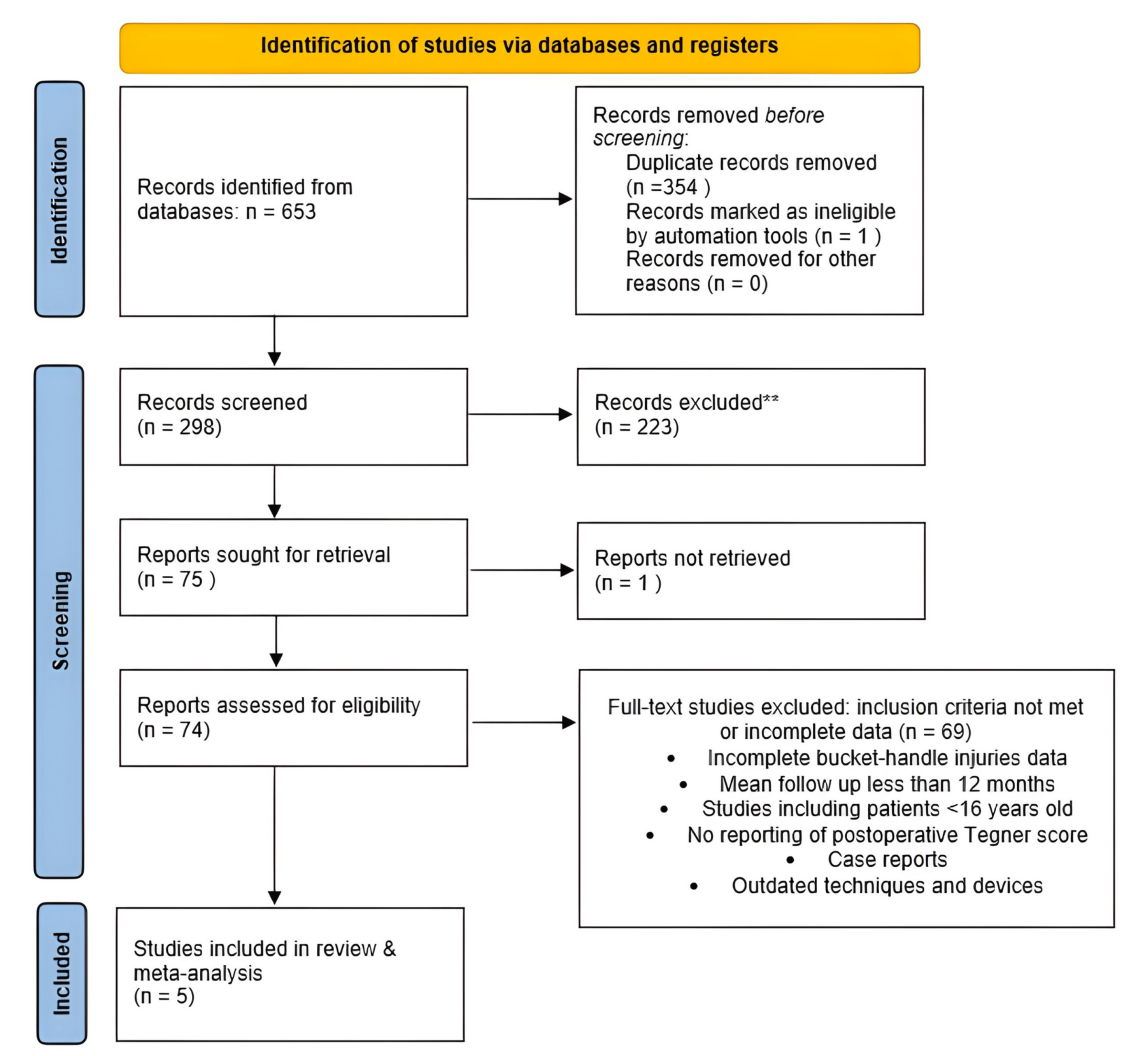
Flow diagram of the study selection according to the PRISMA guidelines. PRISMA: Preferred Reporting Items for Systematic Reviews and Meta-Analyses.

Two researchers (VT, VD) performed the literature search independently. If a consensus was not reached, the reviewers referred to a third author as a referee (TK). The primary search of the databases yielded 653 records. After removing duplicates, 298 studies were selected for abstract review, and 223 studies were consequently selected for a full review. One study was further excluded to minimize the risk of bias due to the use of an outdated repair device. After applying all the eligibility criteria, five studies were included in the meta-analysis. The relevant PRISMA flowchart is presented in Figure 1.

Level of Evidence and Study Quality

Two of the included studies were retrospective cohort studies (level of evidence 3) [4,18], and three were retrospective case series (level of evidence 4) [15,19,20]. After quality assessment with the NOS for case series and cohorts, four studies were assessed as of fair quality and one as of poor quality. Quality assessment is depicted in Table 1.

**Table 1:** Quality assessment of the studies according to the modified Newcastle-Ottawa Scale for case series and cohorts.

Newcastle-Ottawa quality assessment scale for cohort studies					
Quality criteria	Cetinkaya et al. (2018) [15]	Moatshe et al. (2017) [18]	Pelletier et al. (2023) [4]	Uzun et al. (2019) [19]	Yilmaz et al. (2016) [20]
Selection					
1. Representativeness of the exposed cohort	-	-	-	-	-
2. Selection of the non-exposed cohort	-	-	-	-	-
3. Ascertainment of exposure	*	*	*	*	*
4. Demonstration that the outcome of interest was not present at the start	*	*	*	*	*
Comparability					
5. Comparability of cohorts on the basis of the design or analysis	-	*	*	*	*
Outcome					
6. Assessment of outcome	-	-	-	-	-
7. Was the follow-up long enough for outcomes to occur?	*	*	*	*	*
8. Adequacy of follow-up of cohorts	*	*	*	*	*
Total	****	*****	*****	*****	*****
Quality ranking	Poor	Fair	Fair	Fair	Fair

Study and Injury Characteristics

Five studies, with a total of 168 knees with isolated traumatic bucket-handle injuries, were included in the present review. The mean age of the patients was 27.8 years, and the mean follow-up period was 82.4 months. In two studies [4,15], the patient's sex was specified. In one study, all of the six patients were men (100%), while in the other, men comprised 83% of the 122 patients.

Tear Location

One out of five studies [4] reported the location of the tear zone for isolated bucket-handle injuries. Fifty-four (44%) of those tears were located in the red-red zone (Cooper zone 1), 59 (58%) were located in the red-white zone (Cooper zone 2), and nine were located in the white-white zone (Cooper zone 3).

Treatment Options

All the eligible studies surgically treated bucket-handle injuries. Operative treatment included primary repair, using various techniques with different sutures, such as all-inside, inside-out, or outside-in techniques. One study reports the exclusive use of the inside-out technique [18]. One study reported a variety of methods, including all-inside, hybrid, and others [4], and one used the all-inside repair technique [20]. One study used either an all-inside or inside-out technique without further details [15], and one study used a hybrid technique, combining all-inside and inside-out techniques [19].

Reoperations

One study reported three arthroscopic lyses of adhesions in the bucket-handle group. However, the patients of the study that were included in this meta-analysis were a subgroup of non-ACL reconstructed knees. It is not further specified whether those three patients belong in the ACL group or not. Partial meniscectomy is also reported in an isolated bucket-handle tear repair failure. The same study reported one case of infection treated with arthroscopic debridement, partial meniscectomy, and intravenous antibiotic therapy. However, it is unclear whether it was an isolated bucket-handle tear [18]. Another study mentioned meniscectomy as a treatment of failure without further details [4]. None of the studies reported revision meniscal repair.

Rehabilitation

Four out of five studies (156 patients, 92.8%) reported the use of a brace during the early postoperative period [4,15,18,19]. Two studies (23 patients, 13.6%) reported toe-touch weight-bearing protocols [19,20], while one study (17 patients, 10.1%) reported partial weight-bearing [19]. One study (104 patients, 61.9%) reported weight-bearing without further details [4]. The patient demographics and the treatment summary are shown in Table 2.

**Table 2:** Patient demographics and the treatment summary. All values are described as mean and standard deviation (SD). FU: follow-up (months); NA: not applicable. Red-Red meniscal zone, Red-white meniscal zone, and White-white meniscal zone.

Study (year)	Sample size	Sex (%)		Age	Mean FU	Repair technique				Zone		
		Female	Male			Inside-out	All-inside	Hybrid	Other	Red-Red	Red-White	White-White
Cetinkaya et al. (2018) [15]	6	0	100%	25.2 ± 8.8	31 ± 14	NA	NA	NA	NA	NA	NA	NA
Moatshe et al. (2017) [18]	17	NA	NA	32.2 ± 12.25	37.2 ± 12	100%	0%	0%	0%	NA	NA	NA
Pelletier et al. (2023) [4]	122	17%	83%	27 ± 9	98 ± 42	0	67%	32%	1%	44%	48%	8%
Uzun et al. (2019) [19]	11	NA	NA	29.7 ± 8	63.2 ± 12.7	NA	NA	NA	NA	NA	NA	NA
Yilmaz et al. (2016) [20]	12	NA	NA	29.5 ± 7.1	31.3 ± 8.8	0	0	100%		NA	NA	NA
Total	168	NA	NA	27.8 ± 9.3	82.4 ± 44.8	NA	NA	NA	NA	NA	NA	NA

Clinical Outcomes

The summary of failure rates and reported patient outcomes is shown in Table 3.

**Table 3:** Summary of failure rates and reported patient outcomes. All values are described as mean and SD. IKDC: International Knee Documentation Committee; WOMAC: Western Ontario and McMaster Universities Arthritis Index; SF-12 PCS: Short Form Survey Physical Component Summary; SF-12 MCS: Short Form Survey Mental Component Summary; PSS: Patient Satisfaction Scale; Pre-op: pre-operative; Post-op: post-operative; NA: not applicable.

Study (year)	Sample size	Failure rate	Tegner score		Lysholm score		KOOS score		IKDC		WOMAC		SF-12 PCS	SF-12 MCS	PSS
			Pre-op	Post-op	Pre-op	Post-op	Pre-op	Post-op	Pre-op	Post-op	Pre-op	Post-op			
Cetinkaya et al. (2018) [15]	6	16%	2.83 ± 0.75	5.17 ± 2.1	21.17 ± 13.4	84.33 ± 11	NA	NA	53.3 ± 8	74.3 ± 15	NA	NA	NA	NA	NA
Moatshe et al. (2017) [18]	17	0%	NA	5.2 ± 2.4	83 ± 14.3	83 ± 14.3	NA	NA	NA	NA	NA	8.4 ± 9.4	53.2 ± 8.4	54.8 ± 6	6.5 ± 3.5
Pelletier et al. (2023) [4]	122	42%	5.6 ± 2	6 ± 2	NA	NA	43 ± 31	73 ± 31	NA	NA	NA	NA	NA	NA	NA
Uzun et al. (2019) [19]	11	9%	NA	5.7 ± 2.0	47.6 ± 8	88.4 ± 11.3	NA	NA	NA	NA	NA	NA	NA	NA	7.6 ± 2.5
Yilmaz et al. (2016) [20]	12	0%	2.1 ± 1.1	6.9 ± 1.7	70.1 ± 16.2	97.8 ± 3	NA	NA	NA	NA	NA	NA	NA	NA	NA

Postoperative Tegner Score and Return to Sports

**Figure 2:**
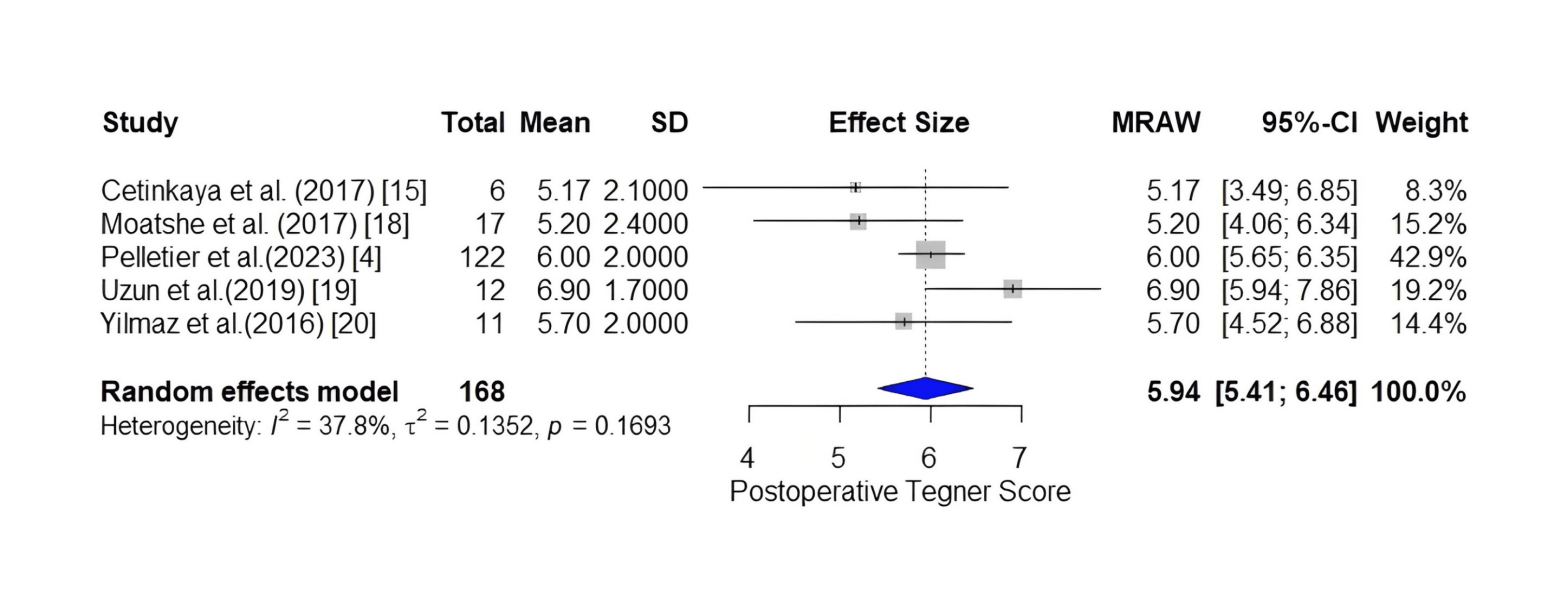
Forest plot of the pooled postoperative Tegner score.

All five studies of this review and meta-analysis reported the postoperative Tegner score, which constitutes the primary outcome of this study. Those scores were incorporated into the meta-analysis. The Higgins and Thompson test returned an I^2^ result of 37.8%, indicating low to moderate heterogeneity between studies. The mean postoperative Tegner score of the meta-analysis was 5.94 (5.41; 6.46), with a 95% confidence interval, indicating a high rate of return to recreational sports among patients with isolated bucket-handle injuries that undergo arthroscopic repair. The relative forest plot is depicted in Figure 2.

Secondary Outcomes

Subsequently, meta-analyses for secondary outcomes were conducted for the mean change in Tegner score among studies, as well as the mean change in the Lysholm score, including studies that reported both the preoperative and postoperative values of the abovementioned scores.

**Figure 3:**
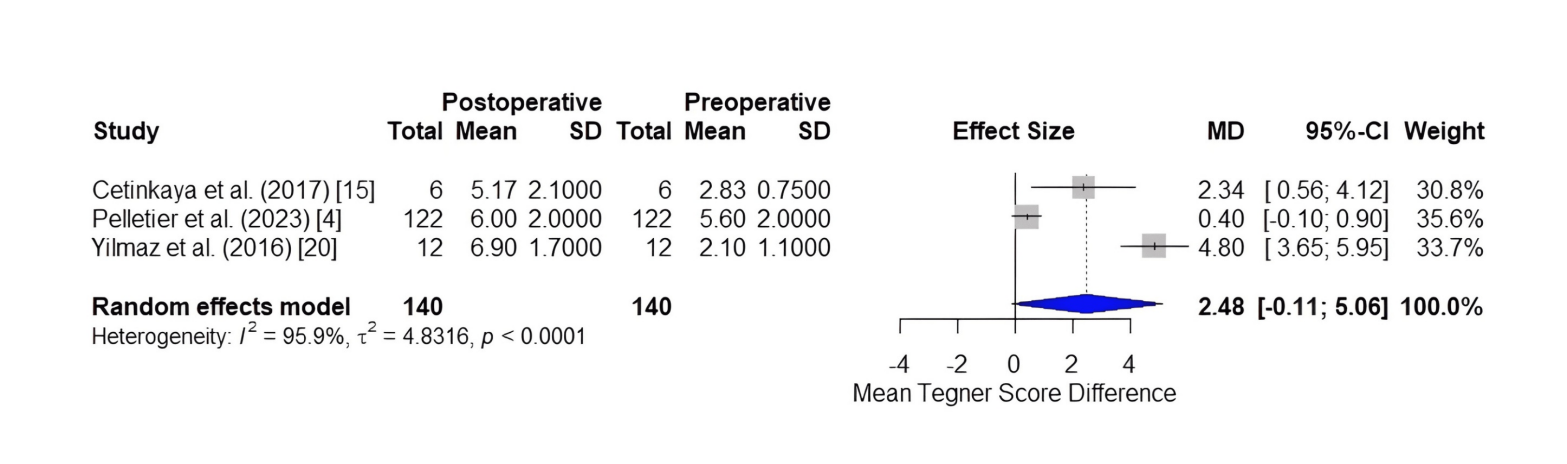
Forest plot of the mean difference in Tegner score.

The preoperative Tegner score was reported in three out of five studies (140 patients). The meta-analysis for the mean change between preoperative and postoperative Tegner score, conducted using the random-effects model due to substantial heterogeneity (95.9%) between the selected studies, returned a mean difference of 2.4782 (-0.1084; 5.0648) (p < 0.0604), a result considered to be statistically insignificant according to the calculated p-value. The forest plot is shown in Figure 3.

**Figure 4:**
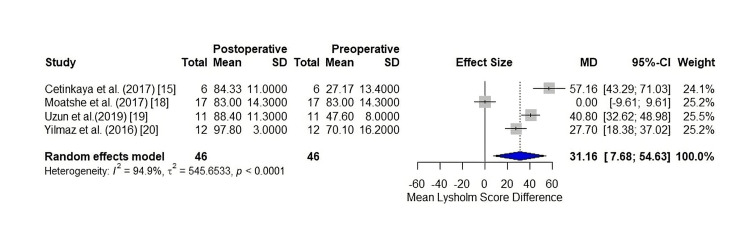
Forest plot of the mean difference in Lysholm score.

The mean change in the Lysholm score according to the random-effects model was 31.16 (7.6799; 54.6343) (p < 0.0093), and statistically significant, with the selected studies for the secondary outcome having substantial heterogeneity (94.9%) between them. The forest plot is shown in Figure 4.

**Figure 5:**
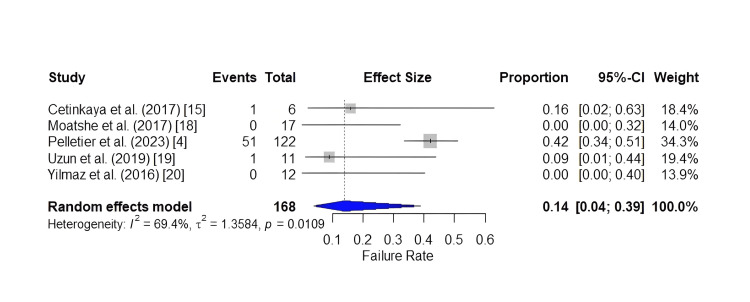
Forest plot of the pooled failure rate.

Pooling of the reported failure rates of the studies included returned a result of 0.14 (0.0405; 0.3884), indicating a 14% (0-42%) pooled failure rate. The forest plot is shown in Figure 5.

Discussion

The main goal of the present study was to evaluate the return to sports following surgical treatment of an isolated bucket-handle injury in the young adult population. To the best of the author’s knowledge, no other systematic review and meta-analysis in the literature currently addresses this topic. The result of this study suggests that most of the included patients had a postoperative Tegner score of 5.41, indicating the ability to perform competitive and recreational sports. However, it should be noted that less than 2% of the patients report a Tegner score of more than 7, indicating the inability to perform highly demanding competitive sports. However, this limitation could be because the studied population comprised non-professional athletes. Moreover, the limited data of the studies on this issue and the percentage and timing of return to sport prevent further conclusions. In addition, the mean difference in the Lysholm score was statistically significant, indicating excellent clinical outcomes despite the substantial heterogeneity of the included studies.

Dzidzishvili et al. [21] systematically reviewed 16 clinical studies involving 1,062 patients treated surgically for bucket-handle meniscal tears with more than two years of follow-up. The study evaluated patient-reported outcome scores and the incidence of failure after repair with either inside-out (IO) or all-inside (AI) techniques. They found that both groups had improved postoperative Lysholm and Tegner scores and that the overall reported failure rate ranged from 6.9% to 20.5% within the AI group and from 0% to 20% within the IO group. More precisely, the mean difference in Tegner score was 1.68, which was statistically significant. This finding is in accordance with the present study, which also demonstrated a significant improvement in the mean Tegner score. However, it is essential to note that Dzidzishvili et al. also included in their study population cases that needed ACL reconstruction, in contrast to the current study population, which consisted of patients with isolated BHMTs.

The included studies followed different rehabilitation protocols but proposed at least six months without sports activity. Yilmaz et al. [20] conducted a retrospective analysis of 52 patients with bucket-handle meniscus tears and repaired them with combined techniques. The patients were started on toe-touch crutch walking for six weeks. After six weeks, the patients were allowed to walk with full weight bearing. The patients were encouraged to return to their routine activity by 12-20 weeks, but sports activity was restricted for six months. An accelerated exercise program was used in meniscus repairs without ACL surgery, with restriction of motion up to 90º for four weeks, no postoperative bracing, and restricted rotational and pivoting movements of the knee and toe-touch weight-bearing for six weeks. In Uzun et al.'s [19] postoperative rehabilitation protocol, patients who had undergone repair of a bucket-handle tear were maintained in a position of full knee extension, using a brace, for the first two weeks after surgery, followed by gradual range of motion into knee flexion. Thereafter, 90 degrees of knee flexion was achieved within four weeks after surgery. From six to 10 weeks after surgery, the patients gradually recovered their maximum range of knee flexion. The patients were allowed to bear weight four weeks after surgery. They gradually recovered their maximum range of knee flexion at six to 10 weeks after surgery and their preinjury activity levels within six months after surgery. In Cetinkaya et al.'s [15] study, during the postoperative rehabilitation period, no flexion was allowed for the first two weeks, with the knee in extension with a brace. The patients were permitted toe-touch weight-bearing with double crutches during this period. Following this, 90-degree flexion and partial weight-bearing exercises were continued until the sixth week. At the end of this period, full flexion without the crutches and full weight-bearing were allowed. Running was initiated by the third month, followed by a return to previous sporting activities in the sixth month.

Two studies used both Barrett's criteria [22] and MRIs to assess clinical failure [19,20], while one study utilized only the Barrett's criteria [15]. In one of the included studies, failure was defined as the need for additional meniscectomy. However, no further details were provided [4]. In contrast, only one study did not specify the criteria applied in their research to assess repair failure [18]. The failure rate reported in recent systematic reviews varies. For instance, Ardizzone et al. [23] included 15 studies with 763 total patients with an average follow-up of 39.8 months, including 396 all-inside BHMT repairs. The overall repair failure rate was 29.3% at an average of 13.0 months. However, it is essential to note that the failure rate decreased to 19.0% for modern devices still in use. Costa et al. [24] performed a systematic review and meta-analysis to determine the failure rate after arthroscopic repair of BHMTs, as reported in the literature. Their analysis included 38 studies with a total of 1358 BHMT repairs. The failure rate of BHMT repairs ranged from 0% to 75%, while the pooled failure rate was 14.8%. In another study by Muench et al. [25], 40 patients with a mean age of 32 underwent arthroscopic repair for acute traumatic BHMT and were followed for at least two years. Revision surgery for arthroscopic partial meniscectomy was performed in 10% of cases before the follow-up visit. The clinical healing rate was 83.3% at the final follow-up. The authors concluded that the patients achieved good to excellent clinical outcomes and a high rate of meniscal healing. Moreover, clinical and radiological healing rates were similarly satisfactory; most patients exceeded the patient-acceptable symptomatic state (PASS) criteria for the International Knee Documentation Committee (IKDC) score. Patients were able to reach a high postoperative activity level. Biological augmentation with platelet-rich plasma (PRP) is also an option to enhance clinical results. However, there is a lack of clear indications on the type of meniscal lesions. Further high-quality clinical studies are needed to support and guide the use of biological strategies for the augmentation of meniscus repair [26].

This research has some limitations. The main ones included are studies with high heterogeneity and low quality. Three studies that reported the preoperative Tegner score showed considerable heterogeneity in the reported outcomes. They also do not further explain whether the preoperative reported score is pre- or post-injury. Most patients in the included studies were part of bigger cohorts containing ACL-reconstructed knees. However, this is usual as current systematic reviews report outcomes on bucket-handle injuries, including in patients with concomitant ACL injuries [21,23,24,27]. In the majority, there were incomplete patient characteristics for the patients with isolated bucket-handle injuries in stable knees. Another significant limitation is the limited number of included studies and the small number of included patients, which may not make the review results reproducible to the general population.

This study highlights the gap in the literature regarding clinical outcomes after isolated bucket-handle tears. There is a need for more extensive patient series and better documentation, as well as higher-level evidence studies, to draw definitive conclusions about return to sports and the long-term clinical outcomes of patients with bucket-handle injuries in knees with ligamentous stability.

## Conclusions

Patients with isolated bucket-handle meniscal tears undergoing surgical repair demonstrate a high rate of return to recreational sports as indicated by the pooled postoperative Tegner score, with significant improvements in Lysholm scores. However, the variability in reporting pre-injury activity levels and return-to-sports timelines limits definitive conclusions. The reported failure rates seem to be consistent across studies, but reflect high between-study heterogeneity and include mixed cohorts with concomitant ACL injuries. The lack of clarity regarding the confounding role of ACL involvement highlights the need for future high-level studies focused exclusively on isolated bucket-handle injuries. Addressing these gaps will improve understanding and guide proper patient management.
